# Letters from Europe

**Published:** 1846-04

**Authors:** James C. Cross

**Affiliations:** Late Professor of the Institutes of Medicine in Transylvania University


### THE WESTERN JOURNAL OF MEDICINE AND SURGERY.

APRIL, 1846.

ART. I.—*Letters from Europe.* By JAMES C. CROSS, M. D., late Professor of the Institutes of Medicine in Transylvania University.

PARIS, December, 1845.

GENTLEMEN:

*Syphilitic Sarcocele.* (Clinic of M. Ricord.)—Syphilitic Sarcocele—Syphilitic Testicle has been the object of much investigation and study, but these do not remount to a very remote period. Astruc had a glimpse of its nature, but he confounded it with blennorrhagic orchitis; Bell was perfectly familiar with it, but Hunter has not even mentioned it. Before the time of Sir Astley Cooper, and even more recently, that of Dupuytren, a satisfactory description of it is not to be found. Syphilitic sarcocele, to which the name of syphilitic albuginitis is still applied, and with reason, for the white tissues are those that are first attacked, is one of the most common and early of the occurrences that are observed in the tertiary period of constitutional pox, and generally attacks one side only.

However, it is not uncommon to see it invade both, either simultaneously or successively. Rarely ever is its existence announced by such symptoms as lead to the apprehension that the testicle will be lost; nevertheless, sometimes in some very rare cases we meet severe, piercing pains, most commonly during the night, in the lumbar region. The patients then complain of a sensation of painful pressure near the kidneys: it seems to them, they say, that a piercing instrument was thrust into the flesh. In some cases the pains assume the character of those that are syphilitic, being experienced during the night, but it is very rare that we find such prelusive symptoms. Generally patients are wholly ignorant of the precise period at which their disease commenced, and commonly it is accident that causes its existence to be made known to them. Sometimes it, however, happens that individuals experience in the testicle a sensation of uneasiness, malaise and dull pain, which directs attention to the part before any appreciable change can be discovered. Commonly the disease commences with a material alteration in the organ diseased without there having been experienced either lumbar or testicular pains. When we attend to the gradual development of the disease the following is what is observed. We find upon a point of the body of the testicle, for it is in the body of the testicle itself that the disease generally commences, and it is but a small portion of the body that it occupies, one or more small indurated nodules;—when the organ is touched carefully a point that resists pressure is felt—which has lost its suppleness—that normal elasticity which is preserved in other parts of the organ. As the disease progresses the resisting points are gradually extended very much in the manner that the bones of the cranium are ossified. In many cases the induration extends in the form of circles, and these in spreading become confounded with one another until ultimately the whole testicle is involved. According as the organ is invaded it loses its elasticity and hardens and concretes. The only difference between what takes place in this affection and inflammation of the testicle consists in the slow and chronic manner of the concretion in the former, while all the phenomena that mark its progress are independent of inflammation. In typical and regular cases the epididymis is not involved in the diseased action, and for a considerable time during the progress of the disease it may be readily enough distinguished from the body of the testicle which progressively hardens and either presents its normal volume or becomes hypertrophied. One of the characteristics of syphilitic testicle is that it preserves its normal size, and this circumstance which was first signalised by M. Ricord, has induced some writers to believe that this organ was becoming atrophied. There takes place here a phenomenon analogous to that which is observed in those cases of cancer known under the name of *cancer rentrant—cancer concentrique.* In this kind of degeneration where it attacks the breast for example, it imparts to the tissues a horny hardness and causes the nipple to be retracted within them. The same mechanism may be observed in this disease, but it should be remarked that most frequently as the alteration progresses hypertrophy of the testicle supervenes and the organ doubles or triples its ordinary volume. But even in those cases where this takes place the development of the organ is confined within certain limits, while some degenerations of the testicle impart to it a most enormous magnitude. In this disease the augmentation of volume has limits, and this it is important to know, as it will be found essential to a precise diagnosis. When there is an increase in the size of the tumor the augmentation is commonly uniform. This is seen when the albuginitis becomes general; the organ preserves its pyriform appearance—the large extremity being below and the summit more or less elongated extends in the direction of the testicular cord. Syphilitic testicle is not knobbed or knotty—its hypertrophy is regular, and should any inequalities in its surface be detected it will be observed that they present a kind of regularity—they are arranged in zones and not mammillated. When the tumor has acquired three or four times the normal volume of the testicle, if it is examined with attention, the epididymis can be no longer recognized, but will present a perfectly uniform appearance. For a long time it was believed that the epididymis was involved in the disease and participated in the alteration which the body of the organ itself experienced. But this is a mistake, for pathological anatomy has shown that it is perfectly healthy and only flattened against the organ. The tissue which envelopes the epididymis is stretched and appropriated by the progressively augmenting testicle and is thus flattened and spread out upon the body of the testicle. Not only does the epididymis remain unaffected by the disease, but the deferant canal also. This is likewise a sign of the highest diagnostic importance, for if there is a degeneration either of the epididymis or the cord we have satisfactory assurance that there is some complication. The tissues of the cord remain perfectly healthy, and the disease may exist in its most exasperated form—the tumor may attain its fullest development without any change in the epididymis and deferant canal—without pain either in the organ or its vicinity, whether the testicle be subjected to pressure or not.

In many patients when the disease is fully developed, there is observed a circumstance that cannot but be regarded as singular, and which is that the exquisite sensibility of the testicle in the normal state is lost. Often the tumor when it becomes voluminous occasions tractant pain in the cord itself, and from which results respondent pain in those parts that sympathise with the testicle. It is maintained by M. Ricord that the pains are not symptomatic, but sympathetic. During the whole course of the affection up to this point the tumor is hard and solid, and when pressed imparts to the fingers the sensation of inelastic fibro-cartilaginous tissues. There is no change in the skin—its color is normal—its temperature nearly natural, and it is mobile, not having formed any subjacent adhesions. In a few rare instances we see a slight enlargement of the veins of the scrotum. We should here speak of a complication which is present sometimes and presents various degrees of development;—allusion is made to hydrocele of the tunica vaginalis. It is not very usual to see this affection complicated with a hydrocele that presents all the characteristics of symptomatic hydrocele. Besides, there is no febrile reaction or even functional disorder except what may be in the diseased organ, and there, as the affection progresses and increases in depth, the spermatic fluid diminishes in quantity and ultimately fails to be secreted altogether. This is more especially the case when both testicles are simultaneously attacked, and then the faculty of erection diminishes, and is at last completely lost. The physical properties of the semen in regard to quantity and consistence undergo very sensible changes. These changes are progressive, and when they are studied with the microscope the following is the order in which they take place:—the spermatic animalcules first disappear; then the spermatic crystals, and finally the result of the testicular secretion becomes nothing more than a transparent fluid, without consistence, and bearing a very strong resemblance to pure water. The progress of syphilitic sarcocele is slow—essentially slow and indolent—it may not only last many months, but even one, two, or three years. What is very remarkable is that in uncomplicated cases, and it may be laid down as a law to which there is no exception, that syphilitic sarcocele never suppurates—it has no tendency to suppuration, and never exceeds a certain volume. When it has arrived at a certain point, nearly the same in all cases, resolution commences, and the course of the affection is directly the reverse of that which has been described. This is sometimes the result of treatment, and sometimes that of a spontaneous effort of the economy. To see the organ gradually restored to its natural size is at first a source of great satisfaction to those afflicted with this disorder, but this is of short duration, for they are soon frightened to see the diminution in volume obstinately persisting, and this continues until the organ disappears entirely.

*Organogenie.*—At a recent meeting of the *Academy of Sciences*, M. Coste read an article entitled—*Rescherches sur les premieres modifications de la matiere organique, ou sur la for-*

*mation des cellules.* We are told that if the tissues of animals are studied in the germ we shall see that they are in great part composed of cells as are those of vegetables. From the moment that it was demonstrated that the cell constituted the base of all the organised tissues, that it is, as it were, their integral molecule, the greatest importance was attached to the mechanism of its formation. M. De Mirbel was the first to institute an inquiry into the manner in which the cell proceeds from the cambium to form its parietes at the expense of this mucilage. When he cut the extremity of a root of the date-tree, he saw in the mucilaginous substance discharged a multitude of irregularly spheroidal masses that evidently resulted from the concentration of the mucilage, which in each condensed mass, exhibited already the first rudiments of a proximate organization. In the centre of each mass there is soon formed a cavity that gradually increases in size by pressing upon the matter by which it is surrounded and which serves to limit it. This matter thus pressed upon is attenuated into the form of a membrane by the dilatation of the central cavity, and ends by representing a hollow sphere which is nothing more than a vesicle. To this theory, M. Coste opposed that of Schleiden, applied by Schwann to the organization of animals, and which is merely a generalization of the theory of Purkinje on the development of the egg in the ovary. Having recognized that the germinative vesicle was the part of the egg which, from the commencement has the greatest proportional development, Purkinje considered it as a centre around which is successively deposited the vitellus and vitelline membrane, which coagulating in its turn at the periphery of the yolk, completed the ovarian egg. This successive encasement of the concentric parts having appeared to Schleiden and Schwann as the most simple means that could be conceived of the formation of the vesicular parietes, they constructed upon it a general theory to explain the development of the vesicle. Before we pronounce upon the respective merits of these theories, let us hear what M. Coste has to say on the subject, as he has promised soon to give us his own views which we doubt not will at least assume to be novel.

*Etiology of Goitre.*—On the same occasion, M. Guyen, Chief Surgeon of the Army in Africa, made a communication on the causes of Goitre. He contends that it cannot with truth be referred to a low temperature—to the nature of the water, or the humidity of the atmosphere. He alleges that the causes assigned are wholly insufficient to explain the production of a disease that prevails in so many places, and which everywhere exhibits the same characteristics. The cause of Goitre, according to M. Guyen, is the imperfect and insufficient exposure to the sun of those localities in which it prevails. In support of this opinion he quotes the observations made by Fodere, that Goitre is not found in elevated situations nor in open plains, but in the deep and narrow gorges of mountains. He explains the presence of this disease in Lyons by referring it to the fact, that that city does not enjoy the benefit of the rays of the rising sun in consequence of a mountain in its vicinity, which runs from the North to the South. Although it may be true that Goitre is prevalent in localities secluded from the full power of the sun, and even more prevalent in such localities than in those differently situated, the theory of M. Guyen is far from satisfactory, for Goitre is common on the Andes—in countries perfectly dry and exposed in the fullest extent to the sun.

*Aneurism cured by Galvano-Puncture.*—M Petrequin has made a publication on this subject, of which the following is a summary analysis. The author states that when we consider the danger which an operation for aneurism involves, any safe means by which we may dispense with it, must be considered a great conquest. The following are some of the results of his analysis of the operations that have been performed for this disease, when affecting the principal arteries. He thinks, one-sixth at least, of those operated on for aneurism of the crural and primitive carotid die; of the external iliac, one-fourth; of axillary and subclavian, one-half; of the branches of the brachio-cephalic trunk, two-thirds, &c. This being the case, we may form some idea of the value of a process not only more simple, but one devoid of danger. The application of electricity, says M. Petrequin, has been suggested, but no advantage has resulted from it, and all that the science furnishes on the subject is to be found embodied in the following lines written by M. M. Marjolin and Berard, in 1833. "They have endeavored to induce a coagulation of the blood in the sac by electricity transmitted through needles plunged into the tumor. *This idea*, which originated with M. Pravaz, has not yet, to our knowledge, been reduced to practice." To satisfy himself on the subject, Petrequin addressed Pravaz, from whom he learned that it was nothing more than a speculative thought which researches on another subject had inspired, and that he had never performed experiments on man or animals to test its practicability. Petrequin experimented upon human blood immediately upon its abstraction, and the results obtained were so satisfactory as to induce him at once to make application of it to the cure of aneurism. The case in which it was first employed and with success, was a traumatic aneurism of the temporal artery of the size of an almond, of a soft consistence and slightly sensible on pressure. Every precaution was taken to determine the precise nature of the tumor, and that it was aneurismal there could not be the least doubt. In the presence of many persons two fine, sharp steel pins were plunged into the tumor about four-fifths of an inch, in such a manner as to cross at right angles. A communication was established between the heads of the pins and the poles of a pile. The first instant of contact produced a shock and severe pain which went on increasing as the galvanism was augmented, until they became so intense as to render it necessary to suspend the sitting. The duration of the galvano-puncture lasted about 10 or 12 minutes, and in that time the direction of the galvanic current was changed three times. During the operation the pulsations progressively diminished, until at length they ceased entirely. The pulsating aneurism was replaced by a solid and hard tumor, which at the expiration of ten days, was being gradually absorbed. This cure was considered radical. Now, M. Petrequin thought that he had fully established the priority of his claim to having first practised galvano-puncture for aneurism when he consulted Pravaz and obtained from him the assurance that he had never tried it; but he was mistaken. At a meeting of the *Academy of Sciences*, on the 8th inst., we were surprised, if not amused, to find that a letter had been received from Mr. B. Phillips, of London, who claims over M. Petrequin, of Lyons, the priority of the employment of galvano-puncture in cases of aneurism. The profession will doubtless rejoice to learn that they are not disputing over the skin of the bear before the bear is killed.

*Chemistry.*—At the meeting of the *Academy of Sciences*, on the 8th inst., we heard, with great interest, a communication read by M. Dumas, on behalf of M. Gehrarde, Professor of the Faculty of Sciences of Montpelier. This article treated of one of the highest and most difficult questions of modern chemistry, and as it professed to have for its object the enlightenment of a point that had engaged the close attention of the ablest men who have made this branch of learning a subject of special study, it could not fail to inspire the greatest interest. His experiments, a detailed account of which was embodied in the communication made by M. Dumas, were instituted to ascertain the atomic weight of Chlorine. When different bodies combine it is admitted that they are composed of molecules or atoms. Chemists have determined the weight of these molecules and have found that they vary in the different elementary bodies, and that the atomic weights of all bodies are exact multiples of hydrogen. That the atomic weight of carbon is exactly six times; that of azote fourteen times; that of sulphur sixteen times that of hydrogen; and now M. Gehrarde asserts the atomic weight of Chlorine is exactly thirty-six times that of hydrogen. This being the case, it is satisfactorily proved that these weights are not arbitrary, but regulated upon a principle that is fixed and universal. Protracted and very carefully instituted researches had been prosecuted by M. Dumas to establish this fact, and he succeeded in showing that all the bodies that had been studied, with the exception of Chlorine, were subordinate to it. This exception, however, was fundamental and threatened the overthrow of the opinion maintained by M. Dumas and his followers, more especially as it rested upon the experiments of such men as Berzelius, Marignac and Pelouze, who always decided its atomic weight to be 35.3 or 35.5, that is to say, a fractional number being neither 35 nor 36. M. Gehrarde, unappalled by the difficulty of the subject, and unalarmed at the danger of a contest with names and researches that are justly honored as classical, examined the processes employed by his illustrious predecessors, and detected a source of error which had escaped their attention. His experiments, which are regarded as decisive, makes the atomic weight of Chlorine 36, and is therefore the exact multiple of the atomic weight of hydrogen. The problem may consequently be considered as solved, and the opinion maintained by M. Dumas and his followers, seems destined to triumph over that of Berzelius, which has so long prevailed

*Amphioxus.*—Some zoologists apply their exclusive attention to the completion of the great catalogue of living beings, and to point out the means by which the species may be distinguished from one another, while others, curious to know the secrets of natnre, have devoted themselves more particularly to anatomical and physiological researches—they have sought to discover how life, considered under the two-fold relation of its manifestations and instruments, is modified in the different animals, and they have therefore directed their observations especially to those points which seem best calculated to reflect light upon the laws of animal organization. Like the cavern of Rasselas, which opens to the most beautiful landscapes, a future, brilliant with the most magnificent discoveries awaits this *ecole physiologique*, of which, in France,

Milne Edwards is the distinguished representative, and to which M. De Quatrefages belongs, to whose researches on the *Amphioxus* we would direct attention. The *Amphioxus* had been already seen by the celebrated Pallas, but having at his disposition animals only which were preserved in alcohol, he took it for a *Limas.* It was found by M. Costa, at Naples, and judging from its external characteristics only, he decided it to be a fish. The fact, however, is that the *Amphioxus* is scarce more of a fish than a limas—it scarcely deserves to be placed in this great branch of the vertebrata, and yet it is impossible to class it amongst the invertebrata. It has, properly speaking, no brain—the cranium is wholly wanting—it has no heart—its blood is white and devoid of the globules so characteristic of the superior animals. To give an account of the relations which connect the whimsical organisation of the Amphioxus with the rest of the animal creation, it seems necessary to admit the truth of some of those ideas which modern science appears daily to render more and more reasonable, but which are obstinately rejected by many able and learned men. In other words, we must admit that the *Amphioxus* is a degenerated vertebral—that while its anatomical structure is simplified it still preserves some of the characteristics of the animals of this type. The characters which persist are found in the vertebræ before they become fully developed, and therefore, if the expression may be used, it may in this respect be denominated a permanent embryo. Already Muller, the great German physiologist, had made a voyage of the Baltic Sea for the express purpose of studying in the living Amphioxus this vertebral, which in so remarkable a manner differs from all others, and he published a work embodying the results of this investigation, which by naturalists is thought to posses very high interest. M. Quetrefages found the Amphioxus at Messina, not far from the famous gulph of Charybdis—he reviewed the observations of Muller—confirmed them on many points, and completed them on others. He detected the existence of several nerves that had escaped the attention of his predecessor—he has verified the existence of real eyes, which he found deeply embedded in the substance of the tissues, and has exhibited the singular structure of what we would call the cellular tissue. We might give more details, but these are sufficient to impart a clear idea of the Amphioxus, while it cannot fail to strike the reflecting physiologists how forcibly its study illustrates how very fallible must be those general conclusions and laws that have been deduced from the study of the superior animals exclusively.

*Variola modified by Vaccination.*—A young man, 18 years of age, was attacked with small-pox in Bouillaud's ward, in the Hospital of Charity, after having experienced for four days only the precursory symptoms. He was vaccinated the first day by means of twelve or fifteen pricks in each arm. The variolic eruption went through its stages with great rapidity and without secondary fever. When desquamation commenced the vaccine disease which had remained in a perfectly latent state, was developed, and in its turn went through its stages with great rapidity, which demonstrated it had also experienced some modification. This remarkable fact proves that when variola attacks persons not vaccinated, vaccination is proper and may be useful if only occasionally, not only during the primary fever, but even at the commencement of the variolic eruption.

*Resuscitation after submersion for half an hour.*—In the *Journal de la Corse* a case is recorded with the utmost minuteness and detail of a woman who was completely resuscitated after being under water for the space of full half an hour. Of the truth of this, from the testimony adduced on the occasion, there could be but little doubt; but as the circumstances of submersion and ultimate recovery are to be found only in a political and not a medical publication we shall not trouble you with the details. The case, however, is in some degree interesting, and may excuse the propounding of some questions. If true, how did she survive through the half hour of submersion? Was it owing to a state of asphyxia which supervened before falling into the water? Was it because the communication between the ventricles of the heart were not completely closed and that the circulation continued as in the fœtus in utero? Is it possible for a human being, after the establishment of extra-uterine life, to live for the space of half an hour without breathing? The case is certainly extraordinary, and, we believe, unique of its kind.

