# Erratum

**Published:** 1846-04

**Authors:** 


*Erratum.*—An error in the paging of this number, which ought to have begun with 277 instead of 253, has escaped the proof-reader; but it will be seen that the error is merely in the paging, no matter being omitted.

